# Super Carbonate Apatite-miR-497a-5p Complex Is a Promising Therapeutic Option against Inflammatory Bowel Disease

**DOI:** 10.3390/ph16040618

**Published:** 2023-04-19

**Authors:** Naoto Tsujimura, Takayuki Ogino, Masayuki Hiraki, Taisei Kai, Hiroyuki Yamamoto, Haruka Hirose, Yuhki Yokoyama, Yuki Sekido, Tsuyoshi Hata, Norikatsu Miyoshi, Hidekazu Takahashi, Mamoru Uemura, Tsunekazu Mizushima, Yuichiro Doki, Hidetoshi Eguchi, Hirofumi Yamamoto

**Affiliations:** 1 Department of Gastroenterological Surgery, Graduate School of Medicine, Osaka University, Yamadaoka 2-2, Suita City 565-0871, Japantogino04@gesurg.med.osaka-u.ac.jp (T.O.); ysekido@gesurg.med.osaka-u.ac.jp (Y.S.); tsuyoshihata@gesurg.med.osaka-u.ac.jp (T.H.); nmiyoshi@gesurg.med.osaka-u.ac.jp (N.M.); htakahashi@gesurg.med.osaka-u.ac.jp (H.T.); muemura@gesurg.med.osaka-u.ac.jp (M.U.); ydoki@gesurg.med.osaka-u.ac.jp (Y.D.); heguchi@gesurg.med.osaka-u.ac.jp (H.E.); 2 Department of Gastroenterological Surgery, Kansai Rosai Hospital, 3-1-69 Inabaso, Amagasaki 660-8511, Japan; 3 Department of Molecular Pathology, Division of Health Sciences, Graduate School of Medicine, Osaka University, Yamadaoka 1-7, Suita City 565-0871, Japanyyokoyama@sahs.med.osaka-u.ac.jp (Y.Y.); 4 Division of Systems Biology, Nagoya University Graduate School of Medicine, Nagoya 466-8550, Japan; 5 Department of Gastroenterological Surgery, Osaka Police Hospital, Osaka 543-0035, Japan; tmizushima@oph.gr.jp

**Keywords:** inflammatory bowel disease, miR-497a-5p, TGF-β, macrophage

## Abstract

The incidence of inflammatory bowel disease (IBD) is increasing worldwide. It is reported that TGF-β/Smad signal pathway is inactivated in patients with Crohn’s disease by overexpression of Smad 7. With expectation of multiple molecular targeting by microRNAs (miRNAs), we currently attempted to identify certain miRNAs that activate TGF-β/Smad signal pathway and aimed to prove in vivo therapeutic efficacy in mouse model. Through Smad binding element (SBE) reporter assays, we focused on miR-497a-5p. This miRNA is common between mouse and human species and enhanced the activity of TGF-β/Smad signal pathway, decreased Smad 7 and/or increased phosphorylated Smad 3 expression in non-tumor cell line HEK293, colorectal cancer cell line HCT116 and mouse macrophage J774a.1 cells. MiR-497a-5p also suppressed the production of inflammatory cytokines TNF-α, IL-12p40, a subunit of IL-23, and IL-6 when J774a.1 cells were stimulated by lipopolysaccharides (LPS). In a long-term therapeutic model for mouse dextran sodium sulfate (DSS)-induced colitis, systemic delivery of miR-497a-5p load on super carbonate apatite (sCA) nanoparticle as a vehicle restored epithelial structure of the colonic mucosa and suppressed bowel inflammation compared with negative control miRNA treatment. Our data suggest that sCA-miR-497a-5p may potentially have a therapeutic ability against IBD although further investigation is essential.

## 1. Introduction

Inflammatory bowel disease (IBD) such as ulcerative colitis (UC) and Crohn’s disease (CD) is an intractable chronic inflammatory disease, and the number of patients is increasing in the world year by year [1,2,3]. Medical treatments such as 5-aminosalicylic acid (5-ASA), corticosteroids, and anti-tumor necrosis factor-α (TNF-α) antibody are first line-therapies against IBD, but remissions and relapses are often repeated [4,5]. In recent years, anti-interleukin 12/23 antibody, JAK inhibitors, and anti-α4β7 integrin antibody emerged as new molecular-targeted drugs [6,7,8,9], but they carry the risk of immunocompromise, allergy and other side effects and they still cannot cure IBD. Therefore, continuous effort to develop novel therapy is required against IBD.

Although the cause of IBD has not been fully clarified, involvement of genetic factors and environmental factors is suggested [10,11,12]. When the barrier mechanism of the intestinal mucosa is destroyed, food residues and intestinal bacteria are phagocytosed by antigen presenting dendritic cell which present antigen to Naïve T cells and induce differentiation into regulatory T lymphocytes (Treg) and inflammatory T lymphocytes (Th17) [13,14]. In IBD patients, Th17 becomes dominant and Treg declines, so that inflammatory cytokines, TNF-α, and interferon-γ (IFN-γ) increase, and an anti-inflammatory cytokine transforming growth factor-β (TGF-β) decreases [15]. It is reported that TGF-β/Smad signal pathway is suppressed in IBD patients [16,17,18]. Smads involved in this pathway are classified into three types: Inhibitory Smads (I-Smad: Smad 6/7) that inhibit the signal pathway, Common mediator Smad (Co-Smad: Smad 4) that forms a complex with Smad 2/3, and Receptor-regulated Smads (R-Smads: Smad 2/3 and others) that activate the signal pathway [19,20]. It is reported that Smad 7 was highly expressed in mononuclear cells at intestinal lamina propria in patients with IBD [17,18,21]. Intestinal macrophages also play an important role in IBD [22,23,24]. It is reported that intestinal-specific macrophages subset CD14^+^ macrophages produce a large amount of inflammatory cytokines IL-23, TNF-α and IL-6, leading to chronic inflammation in Crohn’s disease [25].

MicroRNA (miRNA) is a single-stranded non-cording RNA of 21 to 25 bases MiRNA that binds to the 3′ UTR of the target mRNA to suppress translation, or control gene expression by cleaving mRNA [26,27]. Although limited numbers of siRNA- and miRNA-based therapeutic options have advanced to clinical stages [28,29,30,31,32,33,34,35], venous infusion of nucleic acid medicine is expected as a powerful therapeutic option especially against severe IBD at acute exacerbation. Using IBD models considerable efforts have been made for systemic delivery of various miRNAs [36,37,38,39,40,41,42,43], but it still remains an unsolved clinical challenge mainly due to lack of suitable delivery system. Thus, miRNA and siRNA are rapidly degraded when administered to the blood stream, which made it difficult to supply sufficient amount of nucleic acid to target lesions.

sCA nanoparticle is a pH-sensitive in vivo delivery system for miRNA and siRNA with no significant immune activation based on modified calcium phosphate method [44]. We had previously reported that systemic administration of sCA incorporating siRNA and miRNA showed antitumor effects in various carcinomas and anti-inflammatory effects in IBD model [44,45,46,47,48,49,50,51,52,53,54,55].

A phase II clinical trial showed that oral Smad 7 antisense oligonucleotides improved clinical symptoms in patients with Crohn’s disease [21], but the phase III clinical study was unfortunately discontinued [56]. Some reports suspect insufficient quality of nucleic acid prepared in the phase III study [56,57,58,59]. Unlike antisense oligonucleotides, miRNA can bind to and regulate multiple genes [26,27]. Instead of single molecule targeting, we currently attempted to identify certain miRNAs based on TGF-β/Smad signal activity, which should exert multiple function. Finally, we investigated therapeutic efficacy of miR-497a-5p in mouse dextran sodium sulfate (DSS)-induced colitis using super carbonate apatite as a systemic delivery vehicle.

## 2. Results and Discussion

### 2.1. Selection of microRNAs That Up-Regulate TGF-β/Smad Signal Pathway

Using a public database TargetScan [60] miRbase [61], 18 mmu miRNAs were selected as candidates which may potentially bind and inhibit expression of negative regulators in TGF-β/Smad signal pathway such as Smad 6, Smad 7, SMURF1, SMURF2, LTBP1, TGIF (Supplementary Figure S1, Point 1). Among 18 miRNAs we chose 13 miRNAs which conserve identical sequences also in human species (Supplementary Figure S1, Point 2). Potential binding combination between 3′ UTR mRNA of the negative regulators and mmu miRNAs are summarized in Table 1.

To find certain miRNAs that activate TGF-β/Smad signal pathway, SBE reporter assays were performed using the two different systems (Supplementary Figure S1, Point 3,4). Supplementary Figure S2 shows the principle of this reporter assay. Thus, once Smad 3/Smad 4 binds to SBE together with various transcriptional factors, luciferase signal comes out.

**Figure 1:**
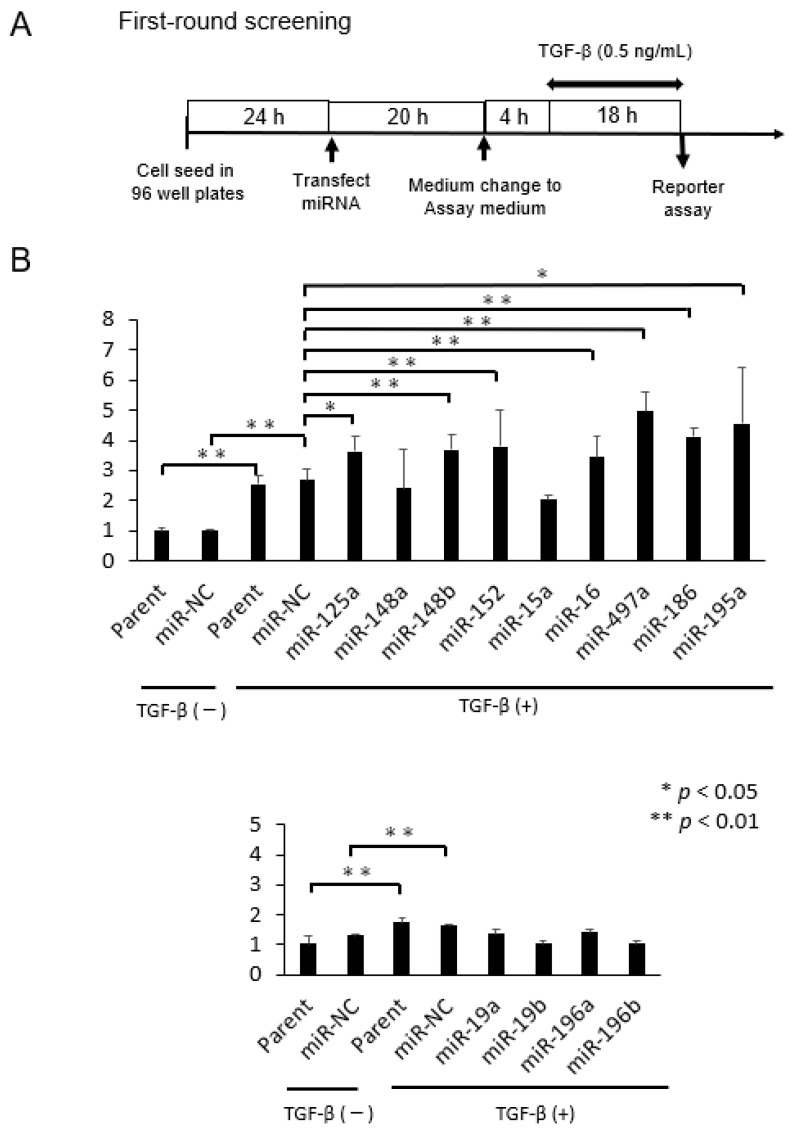
The first-round screening. (**A**) The experimental time schedule is shown here. (**B**) Of the 13 candidate miRNAs, 7 miRNAs significantly activated SBE reporter activity when compared to miR-NC (** *p* < 0.01,* *p* < 0.05, miR-NC vs. miR-125a-5p, *p* = 0.043; miR-NC vs. miR-148b-3p, *p* = 0.005; miR-NC vs. miR-152-3p, *p* = 0.005; miR-NC vs. miR-16-5p, *p* = 0.005; miR-NC vs. miR-497a-5p, *p* = 0.003; miR-NC vs. miR-186-5p, *p* = 0.001; miR-NC vs. miR-195a-5p, *p* = 0.016).

In the first screening, we examined the ability of 13 miRNAs in activation of TGF-β/Smad signal pathway using HEK293 cells where the SBE reporter plasmid was initially transduced. The experimental time schedule is shown in Figure 1A. Thus, cells were exposed with TGF-β at 0.5 ng/mL for 18 h in the assay medium (DMEM supplemented with 0.5% FBS, 1% non-essential amino acids, 1 mM Na pyruvate), and SBE reporter assay was performed. Treatment with TGF-β significantly enhanced SBE activity in parental cells and miR-NC-treated cells (* *p* < 0.05 for each, Figure 1B). We found that 7 of 13 miRNAs activated the TGF-β/Smad signal pathway by TGF-β treatment when compared with miR-NC (* *p* < 0.05, Figure 1B).

**Figure 2:**
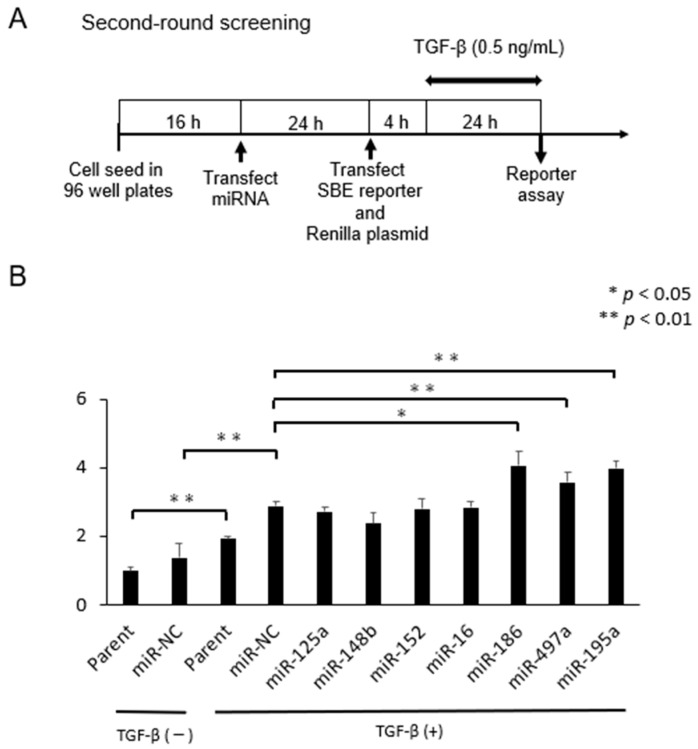
The second-round screening. (**A**) The experimental time schedule is shown here. (**B**) Of the 7 miRNAs, 3miRNAs (miR-497a-5p, miR-186-5p, miR-195a-5p) significantly activated SBE reporter activity when compared to miR-NC (** *p* < 0.01, * *p* < 0.05, miR-NC vs. miR-497a-5p, *p* = 0.016; miR-NC vs. miR-186-5p, *p* = 0.001; miR-NC vs. miR-195a-5p, *p* = 0.001).

In the second screening, we employed a dual luciferase assay system in which SBE activity is normalized by expression of co-transfected Renilla luciferase vector, thus providing more accurate data. Seven miRNAs selected in the first-round screening were transfected 24 h prior to transfection of the plasmids. Then cells were exposed in the assay medium containing 0.5 ng/mL TGF-β for 24 h (Figure 2A). As results, we found that 3 miRNAs (miR-497a-5p, miR-186-5p, miR-195a-5p) again significantly activated the SBE activity when compared with miR-NC (* *p* < 0.05) (Figure 2B). Because miR-195a-5p had already been reported as a potential treatment option for IBD by promoting intestinal barrier integrity and restoration of the intestinal epithelium [62,63], we focused on miR-479a-5p and miR-186-5p in the subsequent experiments.

### 2.2. Effect of miRNA Treatment on Smad Expression

**Figure 3:**
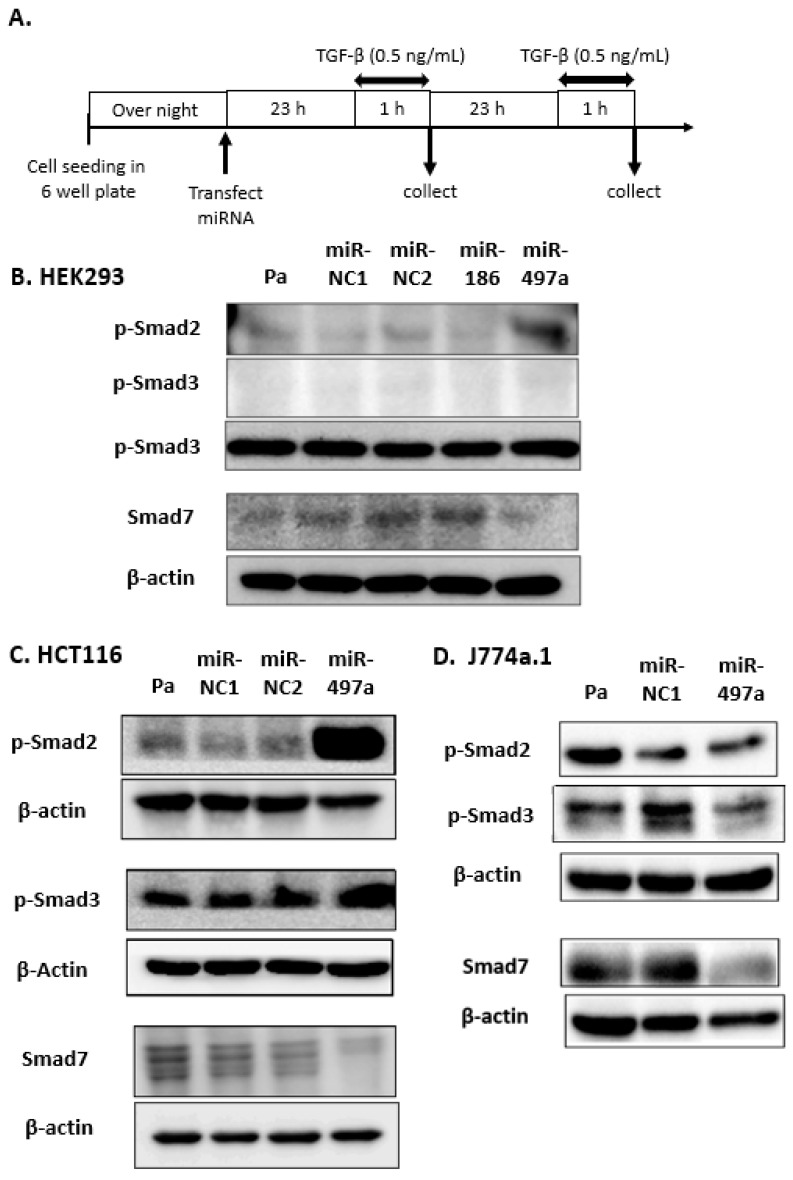
Western blot analyses for Smads in TGF-β/Smad signal pathway. (**A**) Experimental time schedule is shown here. (**B**) In HEK293cells, miR-497a-5p suppressed the expression of Smad 7 and increased the expression of p-Smad 2 48 h after transfection. The expression of p-Smad 3 was not detected. (**C**) In CRC line HCT116, miR-497a-5p suppressed the expression of Smad 7 and increased the expression of p-Smad 2 and p-Smad 3 24 h after transfection. (**D**) In mouse macrophage cell line J774a.1, miR-497a-5p suppressed the expression of Smad 7 miR-NC 48 h after transfection. The expressions of p-Smad 2 and p-Smad 3 were not affected much.

The sequences of miR-186 and miR-497a-5p were conserved between mouse and human species [60]. HEK 293 cells were transfected with miR-NCs, miR-186, and miR-497a-5p, grown for 24 h or 48 h under treatment with TGF-β at 0.5ng/mL for 1 h, as previously reported [17,64,65,66] (Figure 3A). MiR-497a-5p treatment increased the expression of phosphorylated-Smad 2 (p-Smad 2) and decreased Smad 7 expression compared with parental HEK293 cells, miR-NC1, and miR-NC2-treated cells 48 h after transfection (Figure 3B). By contrast, treatment with miR-186 did not affect p-Smad 2 or Smad 7 expression. In colorectal cancer (CRC) cell line HCT116 under TGF-β treatment, miR-497a-5p up-regulated p-Smad 2 largely and p-Smad 3 to some extent, and decreased Smad 7 24 h after transfection (Figure 3C). In mouse macrophage J774a.1 cells, miR-497a-5p treatment decreased Smad 7 expression 48 h after transfection, although p-Smad 2 and p-Smad 3 levels were maintained as well (Figure 3D).

### 2.3. Smad 7 Is a Direct Target of miR-497a-5p

**Figure 4:**
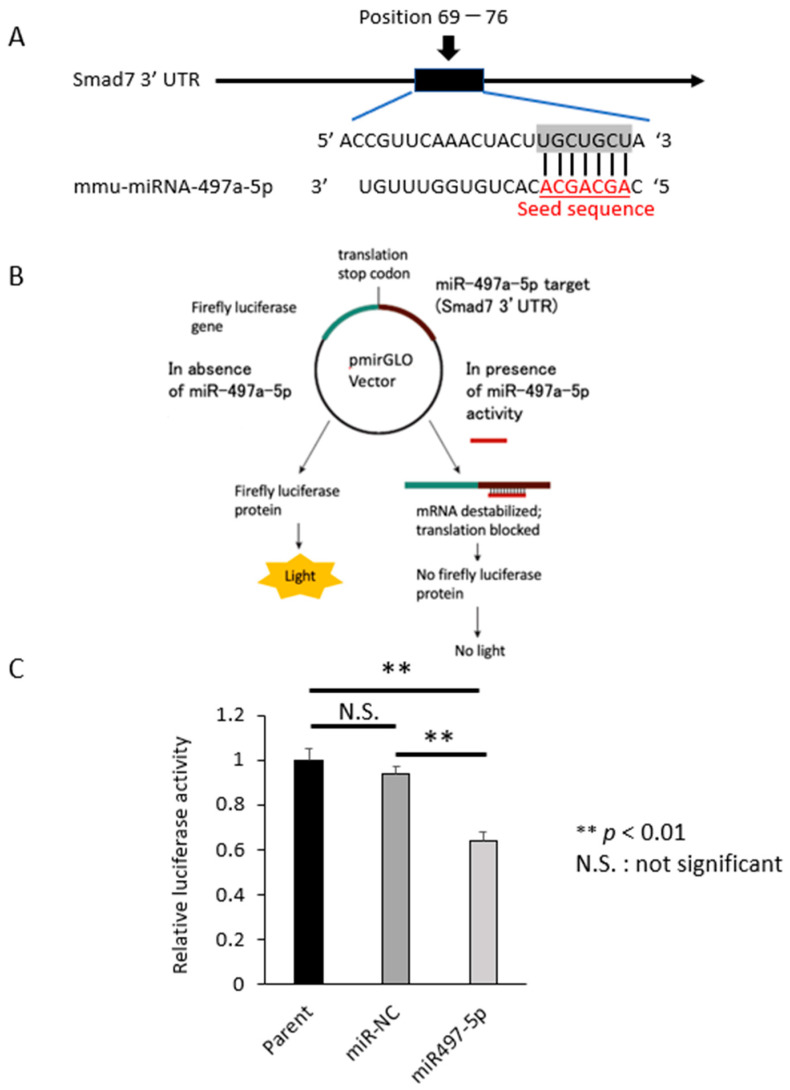
Binding assay of miR-497a-5p and 3′ UTR of Smad 7. (**A**) TargetScan was used to identify a binding site at position 69–76 of the Smad 7 mRNA 3′ UTR that was complementary to the seed sequence of miR-497a-5p. (**B**) Schematic illustration for binding assay. PmirGLO plasmid vector expresses luminescence according to luciferase activity. When miR-497a-5p binds to the cloning site of the 3′ UTR of Smad 7, luciferase luminescence reduces. At 24 h after transfection, firefly and Renilla luciferase activities were measured. (**C**) In CRC cell lines HCT116, miR-497a-5p significantly suppressed the luciferase activities as compared to miR-NC or parental cells (** *p* < 0.01, miR-NC vs. miR-497a-5p, *p* = 0.0007), indicating a direct binding of miR-497a-5p to the sequence of 3′ UTR of Smad 7.

Based on the findings of western blots, we preferentially focused on miR-497a-5p. It is reported that miR-497-5p indirectly activated latent TGF-β via reversion-inducible cysteine-rich protein (Reck) in lung fibrosis model [67]. Here we show that miR-497a-5p directly inhibit Smad 7 expression. In silico survey showed that mouse Smad 7 mRNA has the binding site of miR-497a-5p in its 3′ UTR (Figure 4A). Seed sequence of human miR-497-5p and its binding site in 3′ UTR of human Smad 7 mRNA are both well conserved between mouse and human species (Supplementary Figure S3). We constructed a luciferase reporter plasmid containing the miR-497a-5p binding sites in the 3′ UTR of Smad 7 (Figure 4B). When luciferase assay was performed using HCT116 cells, it was revealed that miR-497a-5p significantly suppressed luciferase activity compared with miR-NC (*p* < 0.05), indicating the direct binding between miR-497a-5p and the 3′ UTR of Smad 7 (Figure 4C).

### 2.4. MiR-497a-5p Suppressed Expression of Inflammatory Cytokines in Mouse Macrophage J774a.1

**Figure 5:**
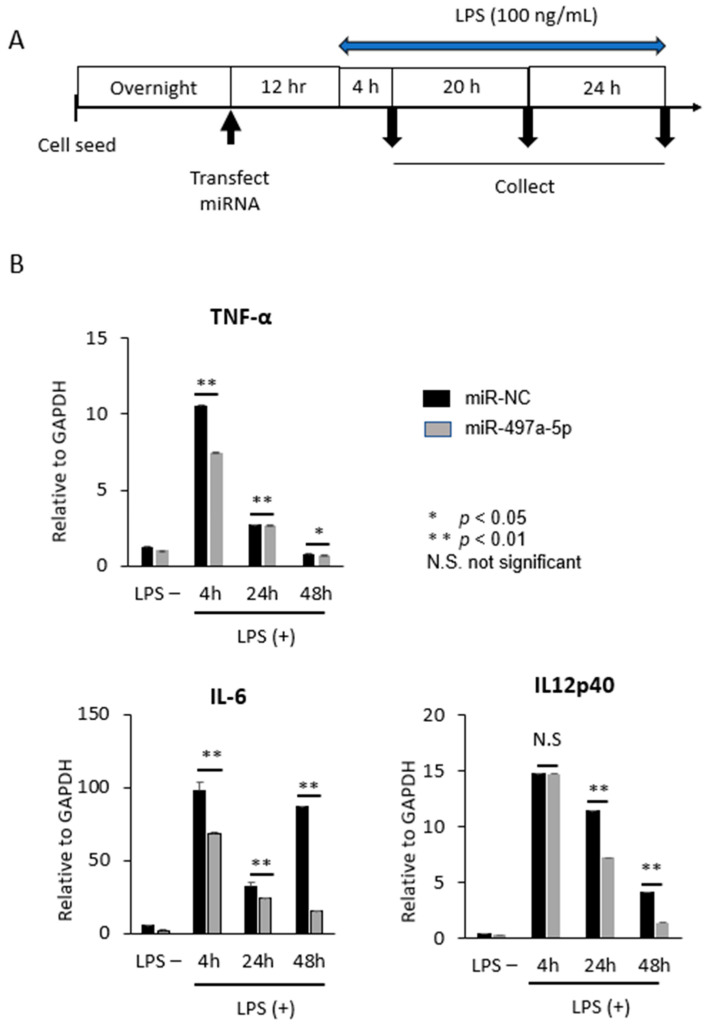
miR-497a-5p suppressed the production of inflammatory cytokine from mouse macrophage cell line J774a.1. (**A**) Time course schedule is shown here. Cells were stimulated by LPS at 100 ng/mL. (**B**) qRT-PCR revealed that miR-497a-5p suppressed the production of inflammatory cytokine, TNF-α, IL-6, and IL-12p40 compared with miR-NC (** *p* < 0.01, * *p* < 0.05. TNF-α: miR-NC vs. miR-497, 4 h *p* = 1.45E-07, 24 h *p* = 0.003, 48 h *p* = 0.033; IL-6: miR-NC vs. miR-497, 4h *p* = 0.0006, 24 h *p* = 0.003, 48 h *p* = 2.36E-09; IL-12p40: miR-NC vs. miR-497, 4 h *p* = 0.496, 24 h *p* = 0.006, 48 h *p* = 0.0005).

It is reported that Smad 7 was highly expressed in mononuclear cells in lamina propria of intestinal mucosa in patients with IBD [16,17,18,21]. A part of mononuclear cells turns into macrophages which produce a large number of inflammatory cytokines such as IL-23, TNF-α, and IL-6 by stimulation of intestinal bacteria, leading to chronic inflammation [23,24,25]. Co-culture of macrophages and intestinal epithelial cells is also used as a colitis model in vitro [68]. Therefore, we examined whether miR-497a-5p would suppress the production of inflammatory cytokines TNF-a, IL-6, and IL-12p40 (a subunit of IL-23), when lipopolysaccharides (LPS) at 100 ng/mL was added to mouse macrophage cell line J774a.1 according to the time schedule shown in Figure 5A. qRT-PCR assays showed that miR-497a-5p suppressed the production of TNF-α, IL-6, and IL-12p40 compared with miR-NC at the indicated time points with asterisks (* *p* < 0.05, Figure 5B).

### 2.5. sCA Delivered miRNA to Macrophages in Colonic Mucosa

**Figure 6:**
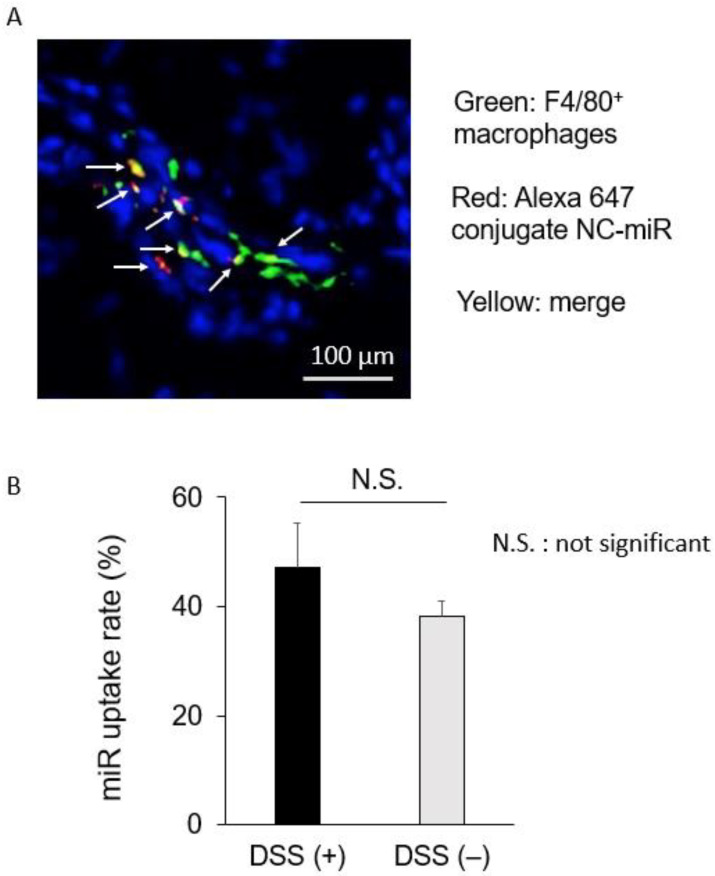
Co-localization of miRNA with macrophages in colonic mucosa. DSS-induced colitis was produced by free drinking of 2% DSS for 7 days in female mice (*n* = 2). sCA incorporating miR-NC tagged with Alexa Fluor 647 (25 μg) was administered via tail vein, and the colon was excised 4 h after administration. Immunostaining of macrophages with the anti-F4/80 antibody showed that co-localization of miRNA with the F4/80 positive-macrophages was noted 47.12 ± 8.27 in inflamed colon by DSS treatment and 38.23 ± 2.79 in normal mucosa, respectively (*n* = 6 per mice). Scale bar, 50 μm. Red: miR-NC tagged with Alexa Fluor 647, Green: F4/80 positive-macrophages. Yellow: merged signals, indicated by arrows.

In our previous study, we showed that sCA incorporating miR-NC tagged with Alexa Fluor 647 was largely co-localized with CD11c^+^ dendritic cells in the inflamed colon [46]. In this study, we performed in vivo uptake test of miRNA into macrophages. To visualize the extent and localization of miRNA in the normal and inflamed colon, sCA incorporating miR-NC tagged with Alexa Fluor 647 was administered via tail vein, and the colon was excised 4 h after administration. Fluorescence microscopy showed that the red fluorescence of the Alexa 647 conjugate miR-NC was present in the mucosa and submucosa of the colonic epithelium. Immunostaining of macrophages with the anti-F4/80 antibody showed that co-localization of miRNA with the F4/80 positive macrophages was often found (Figure 6A) and the percentage of uptake of miRNA in macrophages was 47.12 ± 8.27 in inflamed colon and 38.23 ± 2.79 in normal mucosa, respectively (Figure 6B). There was no significant difference between the two groups.

Anti- TNF-a antibodies such as infliximab and adalimumab are already used in the treatment of IBD [69]. Because miR-497a-5p was able to suppress IL-6 and IL-12p40 in addition to TNF-α in J774a.1, sCA-miR-497a-5p complex targeting macrophages at inflamed colon may have a clinical benefit.

### 2.6. Therapeutic Efficacy of Systemic Administration of sCA-miR-497a-5p on Mouse DSS-Induced Colitis

**Figure 7:**
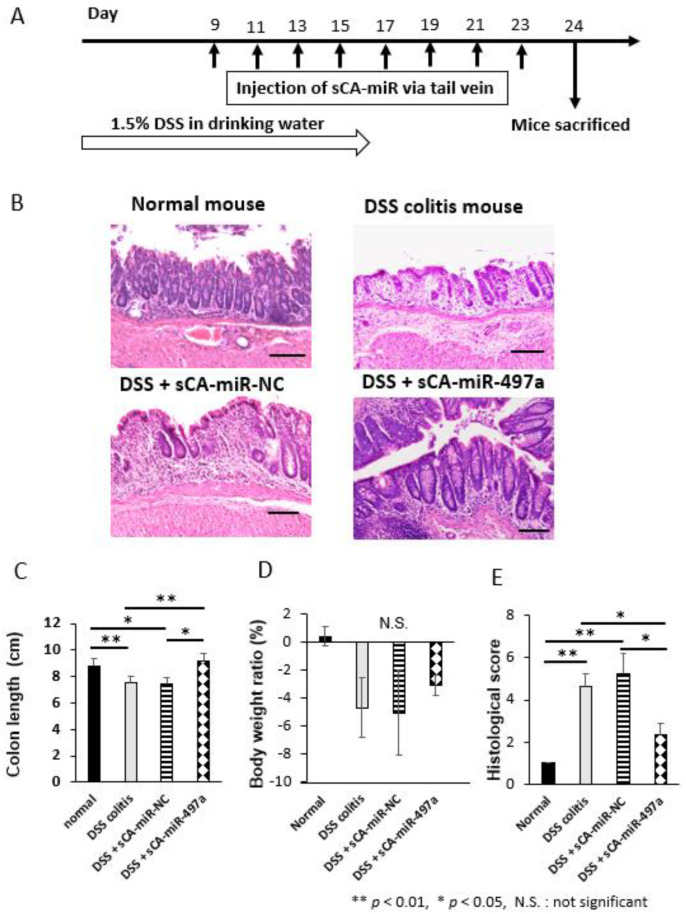
Therapeutic experiment of DSS-induced colitis by intravenous injection of sCA-miR-497a-5p. (**A**) Induction of mouse colitis by 1.5% DSS in drinking water and the therapeutic schedules are shown here. Normal mice (*n* = 3), DSS-treated mice (*n* = 3), DSS and sCA-miR-NC-treated mice (*n* = 4), DSS and sCA-miR-497a-5p-treated mice (*n* = 3). (**B**) H&E staining of distal colon in each group. The mucosal structure was destroyed and many inflammatory cells were noted in DSS-treated mice or DSS and sCA-miR-NC-treated mice. By contrast, DSS and sCA-miR-497a-5p-treated mice had the notable therapeutic effect. Scale bars, 100 μm for each. (**C**) The colon length was significantly longer in mice treated with DSS and sCA-miR-497a-5p as compared to those treated with DSS alone or DSS and sCA-miR-NC (** *p* < 0.01, * *p* < 0.05, DSS alone vs. DSS and miR-497a-5p, *p* = 0.002; DSS and miR-NC vs DSS and miR-497a-5p, *p* = 0.022). (**D**) There was no significant difference in body weight loss among the DSS-treated groups. (**E**) Significantly worse histological scores in mice treated with DSS alone or DSS and sCA-miR-NC were noted, whereas sCA-miR-497a-5p treatment significantly improved the histological damages (** *p* < 0.01, * *p* < 0.05, DSS alone vs DSS and miR-497a-5p, *p* = 0.046, DSS and miR-NC vs DSS and miR-497a-5p *p* = 0.034).

Mice were treated with 1.5% DSS in drinking water for 16 days. sCA-miR complexes were injected to tail vein 8 times on days 9, 11, 13, 15, 17, 19, 21, and 23. On day 24, mice were sacrificed (Figure 7A). Here we attempted a long-term experiment to evaluate the therapeutic efficacy of miR-497a-5p; 1.5% DSS for 16 days followed by therapeutic treatments from day 9 to day 23 every two days. Because most studies were performed to assess preventive effect of drugs or gene manipulation in DSS-induced colitis [69,70,71,72,73], we are not aware of any reports that assessed the therapeutic effect of miRNA in DSS-induced colitis especially in such a long-term schedule. As a result, a drastic inflammatory change was noted as early as on day 5 in the inflamed rectum and colon (Supplementary Figure S4). Compared with normal colon epithelium, DSS treatment alone or DSS and sCA-miR-NC destroyed normal epithelial structures, and numerous inflammatory cells infiltrated into the lamina propria of colonic mucosa (Figure 7B). By contrast, DSS and sCA-miR-497a-5p treatment restored epithelial structures of the colonic mucosa and infiltration of inflammatory cells rather decreased (Figure 7B). The colon length was significantly longer in mice treated with DSS and sCA-miR-497a-5p as compared to those treated with DSS alone or DSS and sCA-miR-NC (* *p* < 0.05, Figure 7C). There was no significant difference in body weight loss among the DSS-treated groups (Figure 7D). Significantly worse histological scores in mice treated with DSS alone or DSS and sCA-miR-NC were noted, whereas sCA-miR-497a-5p treatment significantly improved the histological damages (Figure 7E, * *p* < 0.05).

### 2.7. Therapeutic Efficacy of Systemic Administration of sCA-miR-186-5p on Mouse DSS-Induced Colitis

**Figure 8:**
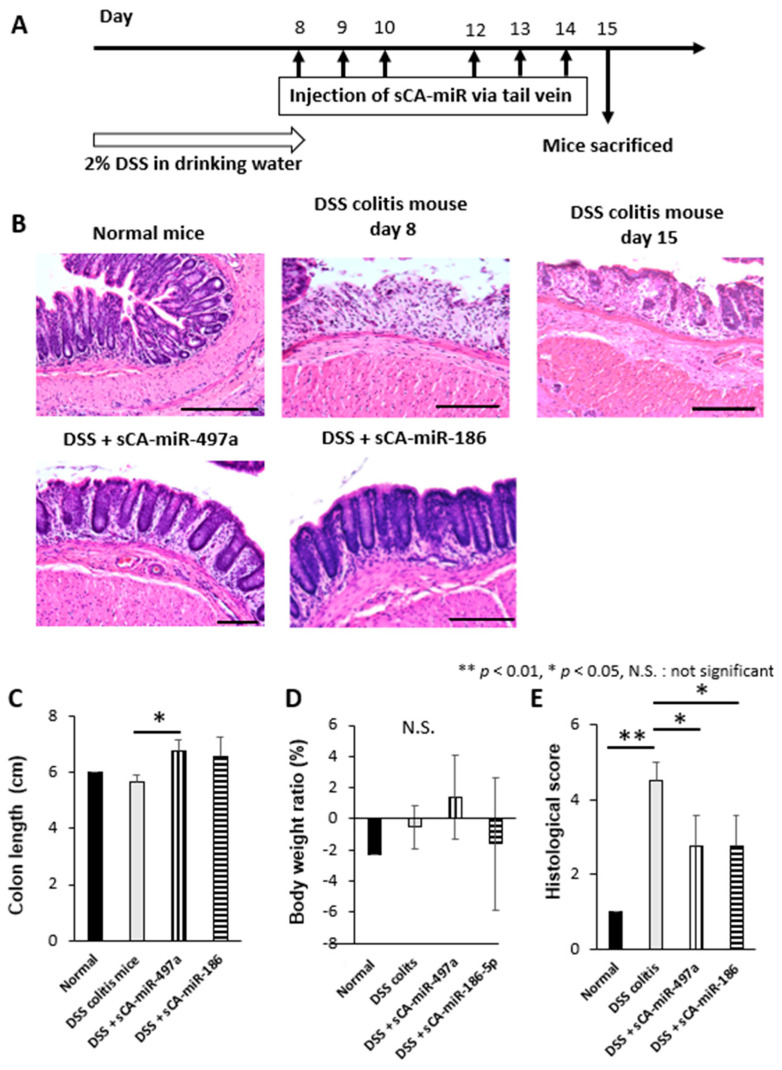
Comparative study on the therapeutic efficacy between sCA-miR-497a-5p and sCA-miR-186-5p. (**A**) The treatment schedule of induction of colitis by DSS and injection of drugs. 2.0% DSS was administered in drinking water for 8 days. sCA loaded with miRNA (50 μg) was injected on days 8,9, 10, 12, 13, and 14. Mice were sacrificed on day 15. The mice were divided into four groups as follows: Normal mice (*n* = 3), DSS-treated mice (*n* = 3), DSS and sCA-miR-497a-5p-treated mice (*n* = 3), and DSS and sCA- miR-186-5p-treated mice (*n* = 3). (**B**) H&E staining. The mucosal structure was destroyed in DSS-induced colitis on day 8, only partially regenerated on day 15. On the other hand, the colonic mucosa was largely reconstructed in DSS and sCA-miR-497a-5p and DSS and sCA-miR-186-5p-treated groups. Scale bars, 100 μm. (**C**) Compared with DSS-induced colitis mice, colon length was significantly longer in DSS and sCA-miR-497a-5p or DSS and sCA-miR-186-5p treatment groups compared with DSS-induced colitis mice (* *p* < 0.05, DSS-induced colitis mice vs. DSS and sCA-miR-497a-5p, *p* = 0.029; DSS-induced colitis mice vs. DSS and sCA-miR-186-, *p* = 0.196). (**D**) Changes in body weight. No significant differences were observed among the groups. (**E**) The histological score was significantly improved in DSS and sCA-miR-497a-5p or DSS and sCA-miR-186-5p-treated mice compared with DSS-induced colitis mice (* *p* < 0.05, ** *p* < 0.01, DSS-induced colitis mice vs DSS and sCA-miR-497a-5p *p* = 0.026; DSS-induced colitis mice vs DSS and sCA-miR-186-5p, *p* = 0.026).

Finally, we compared the in vivo efficacy of miR-186-5p and miR-497a-5p loaded on sCA. Studies have shown anti-tumor effect of miR-186-5p in carcinomas of colon, breast, bladder, prostate, and osteosarcoma through maintaining NK cell stability and suppressing epithelial-mesenchymal transition (EMT) [74,75,76,77,78,79], but its role in IBD has not been investigated. A shorter time course study, where 2% DSS in drinking water was given for 8 days and sCA-miRNAs were injected to tail vein 6 times (Figure 8A), indicated that miR-186-5p had similar therapeutic efficacy to miR-497a-5p in terms of histological score (Figure 8B–E). Our current data with regard to selected three miRNAs acting at activation of TGF-β/Smad signal pathway support the notion that this pathway is an important factor to suppress IBD.

### 2.8. Limitation and Future Perspective

There are several limitations in this study. (i) TGF-β activation and production of cytokines from mouse macrophages had not been examined in the in vivo model yet. (ii) It remains to be clarified how miR-186-5p acts against IBD. (iii) In vivo experiments for miR-186-5p should be repeated although in vivo efficacy of miR-497a-5p was confirmed by two different experiments. During preparation of this manuscript, Zhang M et al. demonstrated a preventive role of miR-497 in DSS-induced colitis using knockout mice and inhibition of Wnt/β-catenin pathway was suggested as one possible mechanism [80]. Collectively it is considered that miR-497 exerts multiple functions such as activation of TGF-β signaling pathway through targeting Smad 7 and inhibition of Wnt/β-catenin pathway. Our study proved therapeutic efficacy of miR-497a-5p using sCA as a delivery tool. Recent review articles introduce sCA nanoparticle as a hopeful non-viral systemic strategy [81,82,83,84].

## 3. Materials and Methods

### 3.1. Cell Lines and Cell Culture

Human colon cancer cell line HCT116 and human embryonic kidney HEK293 cells were obtained from the American Type Culture Collection (Rockville, MD, USA). Mouse macrophage cell line J774a.1 was purchased from JCRB (Japanese Cancer Research Resources Bank) (Ibaragi, Osaka, Japan). HCT116 and J774a.1 cells were cultured in Dulbecco’s modified Eagle medium (Sigma-Aldrich, Cat. No. D6404, St. Louis, MO, USA) supplemented with 10% fetal bovine serum (FBS), 100 U/mL penicillin, and 100 μg/mL streptomycin at 37 °C. HEK293 cells were cultured in DMEM supplemented with 10% FBS, 1% non-essential amino acids (Hyclone, Cat. No. SH30238.01, Tokyo, Japan), 1 mM Na pyruvate (Hyclone, Cat. No. SH30239.01), and 100 U/mL penicillin, and 100 μg/mL streptomycin. Cells were cultured in a humidified incubator at 37 °C in an atmosphere containing 5% CO2.

### 3.2. miRNAs

The specific miRNAs (mmu miR-125a-5p, mmu miR-148a-3p, mmu miR-148b-3p, mmu miR-152-3p, mmu miR-15a-5p, mmu miR-16-5p, mmu miR-497a-5p, mmu miR-186-5p, mmu miR-195a-5p, mmu miR-19a-3p, mmu miR-19b-3p, mmu miR-196a-5p, and mmu miR-196b-5p), and the two negative control miRNAs (NC-miR-1and NC-miR-2) were used in in vitro experiments.

The specific miRNAs (mmu miR-497a-5p) and the negative control miRNA-1 (NC-miR-1) were used in in vivo experiments. The specific miRNAs and NC-miR-1 were purchased from Gene Design Inc. (Ibaragi, Osaka, Japan) and NC-miR-2 was purchased from Sigma-Aldrich. The sequences of miRNAs used are listed in Supplementary Table S1.

### 3.3. TGF-β Pathway-Responsive Reporter Assays

The first round screening was performed using HEK293 cells where SBE reporter plasmid was introduced (BPS Bioscience, Cat. No. 60653, Court West, Suite E San Diego, CA, USA). The cells were maintained with 400 μg/mL of Geneticin (Invitrogen, Cat. No. 10131035, Carlsbad, CA, USA). Cells were seeded in 96-well plates at a density of 2.5 × 10^4^ per well and transfected with miR-NC and candidate miRNAs at a final concentration of 50 nM. The second round screening was performed using SBE Reporter Kit (BPS Bioscience, Cat. No. 60654). The kit contains transfection-ready SBE luciferase reporter vector. This reporter contains a firefly luciferase gene under the control of multimerized SBE responsive element located upstream of a minimal promoter. The SBE reporter is premixed with constitutively expressing Renilla-Sea Pansy luciferase vector that serves as internal control for transfection efficiency. Luciferase assay was performed using Dual-Luciferase^®^ Reporter Assay System (Promega, Cat, No. E1910, Madison, WI, USA) and luminescence was measured by a luminometer (TriStar^2^ LB942).

### 3.4. Transfection

Plasmid DNAs were transfected by Lipofectamine^TM^ 2000 Transfection Reagent (Invitrogen, Cat, No. 11668019) and miRNAs were transfected by Lipofectamine^TM^ RNAiMAX Transfection Reagent (Invitrogen, Cat, No. 13778150). At transfection, Opti-MEM™ I Reduced Serum Medium (Thermo Fisher Scientific, Cat. No. 31985062, Wilmington, DE, USA) was used.

### 3.5. Western Blotting

Cells were seeded in six-well plates at a density of 1 × 10^5^–2 × 10^5^ per well and transfected with miR-NC, miR-497a-5p and miR-186-5p at a final concentration of 50 nM. After 24 h and 48 h, cell lysates were extracted by lysis buffer (0.05 M Tris-HCl pH8.0, 0.15 M NaCl, 0.5 % Nonidet P-40) with 1% proteinase inhibitor cocktail (Nacalai Tesque, Inc. Kyoto, Kyoto, Japan. Cat, No. 04080-24). The protein samples (30 μg/lane) were electrophoresed by SDS-PAGE using 9% acrylamide gel and transferred to PVDF transfer membranes (Bio-Rad Laboratories, Inc. Hercules, CA, USA. Cat, No. #1620177). The membranes were blocked with 5% non-fat dry milk (Cell Signaling Technology, Inc. Cat, No. #9999, Beverly, MA, USA) in TBS with Tween-20 (TBS-T; 50 mM Tris, 158 mM NaCl, 2.7 mM KCl, pH 7.5, 0.1% Tween-20) or Blocking One (Nacalai Tesque, Inc. Cat, No. 03953-66) or Blocking One-p (Nacalai Tesque, Inc. Cat, No. 05999-84) for 1 h at room temperature and incubated with the following primary antibodies overnight at 4 °C:

Antibodies and dilution used were as follows:

Phospho-Smad 2 (Ser465/467) (138D4) Rabbit mAb (1: 1000, Cell Signaling Technology, Cat, No. #3108,), Phospho-Smad 3 (Ser423/425) (C25A9) Rabbit mAb (1: 1000, Cell Signaling Technology, Cat, No. #9520), Smad 7 Polyclonal Antibody (1: 500, Invitrogen, Cat, No. 10466413), β-Actin (13E5) Rabbit mAb (1: 3000, Cell Signaling Technology, No. #4970), and anti-Rabbit IgG, HRP-Linked Whole Ab Donkey secondary antibody (1: 3000, GE Healthcare, Cat, No. NA934, Chicago, IL, USA). The bands were visualized by the ECL Detection System (GE Healthcare Life Sciences, Cat, No. 89168-782) and analyzed using ImageJ 1.52v software (National Institutes of Health).

### 3.6. Binding Assay Using pmirGLO Plasmid Vector

RT-PCR was performed to amplify parts of the 3′ UTRs of Smad 7 miRNA. The primer sequences were as follows: insert of Smad 7, forward 5 5′-GCTCGCTAGCCTCGACTGAGCAGGCCACACTTCAAAC-3′, reverse 5′-ATGCCTGCAGGTCGAGGTGTCCTGCCGATCATACCTG-3′. The amplified product (304 bp) was subcloned and ligated into the multi-cloning site between Sal I and Xho I in the pmirGLO Dual-Luciferase miRNA Target Expression Vector (Promega, Cat, No. E1330) using the In-Fusion HD Cloning Kit (Clontech, Cat, No. 639650, Mountain View, CA, USA).

The sequences of inserts and vectors were confirmed by Sanger sequencing.

Cells were seeded in 96-well plates at a density of 1 × 10^4^ cells per well and were co-transfected with 50 ng pmirGLO plasmid vector containing the insert and either miR-negative control (5 pmol) or miR-497a-5p (5 pmol). At 24 h after transfection, firefly and Renilla luciferase activities were measured using the Dual- Luciferase Reporter Assay System (Promega, Cat, No. E1910). All experiments were conducted in triplicate.

### 3.7. qRT-PCR

Total RNA was extracted using TRIzol^TM^ Reagent (Invitrogen, Cat, No. 15596018). RNA quality was assessed with a NanoDrop ONE spectrophotometer (Thermo Fisher Scientific, Wilmington, DE, USA). About 2 μg of RNA was reverse transcribed with the high-capacity RNA to cDNA Kit (Applied Biosystems, Cat, No. 4388950, Foster City, CA, USA).

qPCR analysis was performed using THUNDERBIRD SYBR qPCR Mix (TOYOBO LIFE SCIENCE, Cat, No. QPS-201). The qPCR was performed on the LightCycler^®^ 480 real-time PCR system (Roche Diagnostics, Basel, Switzerland). The qPCR conditions were as follows: 95 °C for 30 s; followed by 40 cycles of 95 °C for 10 s, 60 °C for 10 s and 72 °C for 30 s. The expression of the target gene was normalized to endogenous GAPDH expression. Relative expression was quantified by the 2^−ΔΔCq^ method.

The primers used were as follows:

TNF-α: 5′-CGTCAGCCGATTTGCTATCT-3′ (forward) and 5′-CGGACTCCGCAAAGTCTAAG-3′ (reverse).
IL-6: 5′-AGTTGCCTTCTTGGGACTGA-3′ (forward) and 5′-CAGAATTGCCATTGCACAAC-3′ (reverse).
IL-12p40: 5′-AGGTGCGTTCCTCGTAGAGA-3′ (forward) and 5′-AAAGCCAACCAAGCAGAAGA-3′ (reverse).
GAPDH: 5′-AGGTCGGTGTGAACGGATTTG-3′ (forward) and 5′-TGTAGACCATGTAGTTGAGGTCA-3′ (reverse).

### 3.8. Therapeutic Model for DSS-Induced Mouse Colitis

Eight-week-old BALB/c mice (female) which retain intact immune system were purchased from CLEA (Tokyo, Japan). DSS (MW 36,000–50,000) was purchased from MP Biomedicals (Cat, No. 9011-18-1, Santa Ana, CA, USA). For producing therapeutic model of DSS-induced colitis, drinking water at a concentration of 1.5% DSS was given to mice for 16 days with reference to previous studies [46,81]. MiR-497a-5p loaded on super carbonate apatite nanoparticle was injected eight times on the tail vein from day 9 to day 23 every two days. Mice were sacrificed on day 24. For a comparative therapeutic study between sCA-miR-497a-5p and sCA-miR-186-5p, 2% DSS in drinking water was given to mice for 8 days [82,83,84]. MiR-497a-5p or miR-186-5p loaded on super carbonate apatite nanoparticle was injected on days 8, 9, 10, 12, 13, and 14. Mice were sacrificed on day 15. The study protocol was in accordance with the Declaration of Helsinki, and the Ethical Guidelines for Medical and Health Research Involving Human Subjects in Osaka University. Animal experiments were approved by the Institutional Animal Care and Use Committee of Osaka University Graduate School of Medicine and by the Committee for the Ethics of Animal Experiments of Osaka University (Permit Number: 30-02-5, 20 June 2018).

### 3.9. Histological Inflammation Scoring of DSS Colitis Mice

Based on previous reports [46,69], the extent of inflammation in colon and intestinal wall was scored as follows: Mucosal damage: 0, normal; 1, focal damage and 3–10 intraepithelial lymphocytes (IELs)/high power field (HPF); 2, rare crypt abscesses plus >10 IELs /HPF; 3, multiple crypt abscesses and erosion/ulceration plus >10 IELs /HPF. Submucosal damage: 0, normal or widely scattered leukocytes; 1, focal aggregates of leukocytes; 2, diffuse leukocyte infiltration with expansion of the submucosa; 3, diffuse leukocyte infiltration. Muscularis damage: 0, normal or widely scattered leukocytes; 1, widely scattered leukocyte aggregates between muscle layers; 2, leukocyte infiltration with focal effacement of the muscularis; 3, extensive leukocyte infiltration with transmural effacement of the muscularis.

### 3.10. Production of sCA

sCA was prepared as described previously [44]. Briefly, 50 μg miR-497a-5p or miR-negative control 1 (NC1) was incubated in 25 mL of inorganic solution (44 mM NaHCO_3_; 0.9 mM NaH_2_PO_4_; 1.8 mM CaCl_2_ pH 7.5) at 37 °C for 30 min. The solution was centrifuged at 12,000 rpm for 3 min. The pellets from two tubes were dissolved in 200 μL saline containing 0.5% albumin, and sonicated (38 kHz, 80 W) in a water bath for 10 min. Approximately 50 μg miRNA per one administration was injected into the tail vein.

### 3.11. Fluorescent Immunostaining of Macrophages at Propria Muscularis of Colon Mucosa

DSS-induced colitis was produced by free drinking of 2% DSS for 7 days in female mice (*n* = 2). Non-treated mice (*n* = 2) served as a comparative reference. The Alexia 647-tagged NC-miRNA (25 μg) encapsulated in sCA was injected into the tail vein and the distal colon was collected 4 h later, and frozen in OCT compound. About 8 μm sections were cut and fixed in 4% paraformaldehyde. The frozen sections (*n* = 6 per mouse) were incubated overnight with rat anti-mouse F4/80 antibody (BIO RAD, Cat, No. MCA497G, Hercules, CA, USA) at a concentration of 1:100. As a secondary antibody, FITC-conjugated goat anti-rat IgG was used (Jackson ImmunoResearch, Cat, No. 112-095-167, West Grove, PA, USA). The nuclei were stained with ProLong Gold anti-fade reagent with DAPI (Invitrogen, Cat, No. #8961). Sections were observed using a fluorescence microscope (BZ-X 700, Keyence Corporation, Osaka, Japan).

### 3.12. Statistics

F-test was performed to find out if there were equal variances between the two groups. Statistical significance of the difference between two groups was then calculated by Student’s *t*-test or Welch’s *t*-test, and data are presented as means ± standard deviations unless specifically otherwise indicated. When more than two groups were compared, one-way ANOVA was used followed by Bonferroni correction to determine the statistical significance of the differences. Statistical analyses were performed using the JMP13 program (SAS Institute, Cary, NC, USA). Differences with *p* < 0.05 were considered significant (File S1).

## 4. Conclusions

In conclusion, we have demonstrated that sCA-miR-497a-5p complex exerts a potent anti-inflammatory effect through activation of TGF-β/Smad signal pathway and inhibition of secretion of inflammatory cytokines from macrophages in IBD therapeutic mice model. These results suggest that sCA-miR-497a-5p may potentially have a therapeutic ability against IBD although further investigation is essential.

## Figures and Tables

**Table 1:** Potential binding combination between inhibitors of TGF-β/SMAD signal pathway and mmu miRNAs.

Gene	mmu miRNA	Position in the UTR	Seed Match Count
SMURF1	125a-5p	2315–2322	8mer
	125b-5p	2315–2322	8mer
	15a-5p	2628–2634	7mer-m8
	15b-5p	2628–2634	7mer-m8
	16-5p	2628–2634	7mer-m8
	19a-3p	642–649	8mer
	19b-3p	642–649	8mer
SMURF2	497a-5p	205–211	7mer-1A
	322-5p	205–211	7mer-1A
	15a-5p	205–211	7mer-1A
	15b-5p	205–211	7mer-1A
	16-5p	205–211	7mer-1A
	195a-5p	205–211	7mer-1A
	19b-3p	2572–2578	7mer-m8
	19a-3p	2572–2578	7mer-m8
	148a-3p	2574–2580	7mer-m8
	152-3p	2574–2580	7mer-m8
	186-5p	2441–2447	7mer-m8
LTBP1	152-3p	37–43	7mer-m8
	148a-3p	37–43	7mer-m8
	148b-3p	37–43	7mer-m8
SMAD6	196b-5p	102–108	7mer-1A
	196a-5p	102–108	7mer-1A
	186-5p	248–254	7mer-m8
SMAD7	15a-5p	69–76	8mer
	497a-5p	69–76	8mer
	195a-5p	69–76	8mer
	15b-5p	69–76	8mer
	16-5p	69–76	8mer
	322-5p	69–76	8mer
TGIF	19a-3p	625–632	8mer
	19b-5p	543–549	7mer-1A
	6965-5p	192–198	7mer-m8
	7075-5p	195–202	8mer
	148b-3p	126–132	7mer-m8
	148a-3p	126–132	7mer-m8
	15a-5p	1709–1715	7mer-m8
	16-5p	1709–1715	7mer-m8
	152-3p	1678–1685	8mer
	195a-5p	1709–1715	7mer-m8
	322-5p	1709–1715	7mer-m8
	497a-5p	1709–1715	7mer-m8

All mmu miRNAs except for miR-15b-5p-, miR-125b-5p, miR-322-5p-, miR-6965-5p, miR-7075-5p are identical to human hsa miRNAs.

## Data Availability

Data is contained within the article and Supplementary Materials.

## Supplementary Materials

The following supporting information can be downloaded at: https://www.mdpi.com/article/10.3390/ph16040618/s1, Figure S1: Screening for candidate miRNAs by SBE reporter assays.; Figure S2: Principle of SBE reporter assay; Figure S3: Sequence of mmu-miR-497a-5p and has-miR-497-5p; Figure S4: HE staining of 1.5% DSS induced colitis mice; Table S1: Sequences of the miRNAs used in this study; File S1: Summary of statistics.
