# Supplementary material for: Super Carbonate Apatite-miR-497a-5p Complex Is a Promising Therapeutic Option against Inflammatory Bowel Disease
Source: Pharmaceuticals (Basel). 2023 Apr 19;16(4):618. doi: 10.3390/ph16040618 (PMC10146939; doi:10.3390/ph16040618)
Supplement: Supplementary file 1 [file pharmaceuticals-16-00618-s001.zip › Figure S1.pdf]

Figure S1. Screening for candidate miRNAs by SBE reporter assays.

1. Through database survey, we picked up 18 miRNA candidates which potentially bind to and suppress the TGF-β signal inhibitors, Smad 7, Smad 6, Smurf1, Smurf 2, TGIF, and LTBP1. **2**. They were then narrowed to 13 miRNAs with respect to identity of miRNAs between mouse and human species for the ultimate purpose of clinical application. **3**. Using HEK293 cells we measured SBE activity which reflects TGF-β/Smad signal pathway through two round reporter assays; the first reporter assay was performed using a HEK293 where SBE reporter plasmid was initially transduced. **4**. In the second reporter assay, we employed a dual luciferase assay system in which SBE activity is normalized by expression of co-transfected Renilla luciferase vector, thus providing more accurate data. Among them, 3 miRNAs (miR-497a-5p, -195a-5p, -186-5p) were eventually selected as the key factors which activated the TGF β/Smad signal pathway.

1. Search for candidate mmu miRNAs by Target Scan and miRbase

18 miRNAs which potentially bind and suppress TGF-β signal inhibitors (SMAD6, SMAD7, Smurf1, Smurf2, LTBP1, TGIF)

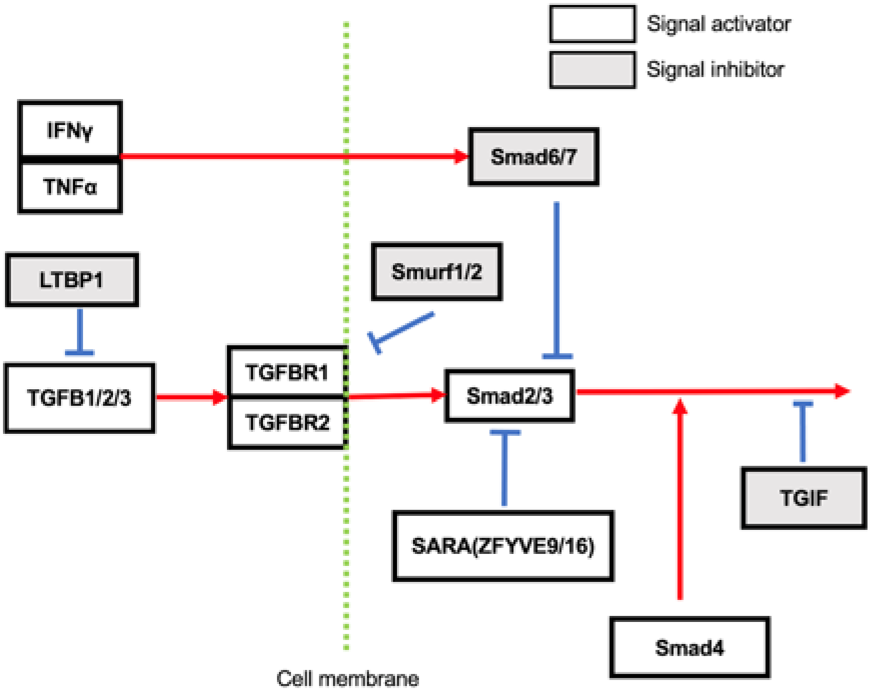

2. Conserved miRNA sequences in human

13 miRNAs retain identical sequences between mouse and human.

3. 1st - round screening: 9 miRNAs were selected

HEK293 cells used, where SMAD binding element (SBE) reporter plasmid is initially transduced(BPS Bioscience, Cat. No. 60653).

⇩

4. 2nd - round screening: 3 miRNAs remained.

HEK293 was transfected by SBE reporter ((BPS Bioscience, Cat. No. 60654) together with Renilla luciferase vector for normalization of transfection efficiency.
