# Supplementary material for: Super Carbonate Apatite-miR-497a-5p Complex Is a Promising Therapeutic Option against Inflammatory Bowel Disease
Source: Pharmaceuticals (Basel). 2023 Apr 19;16(4):618. doi: 10.3390/ph16040618 (PMC10146939; doi:10.3390/ph16040618)

Supplementary Figure S2. Principle of SBE reporter assay.
Once Smad 3/Smad 4 binds to SBE together with various transcriptional factors, luciferase signal comes out.

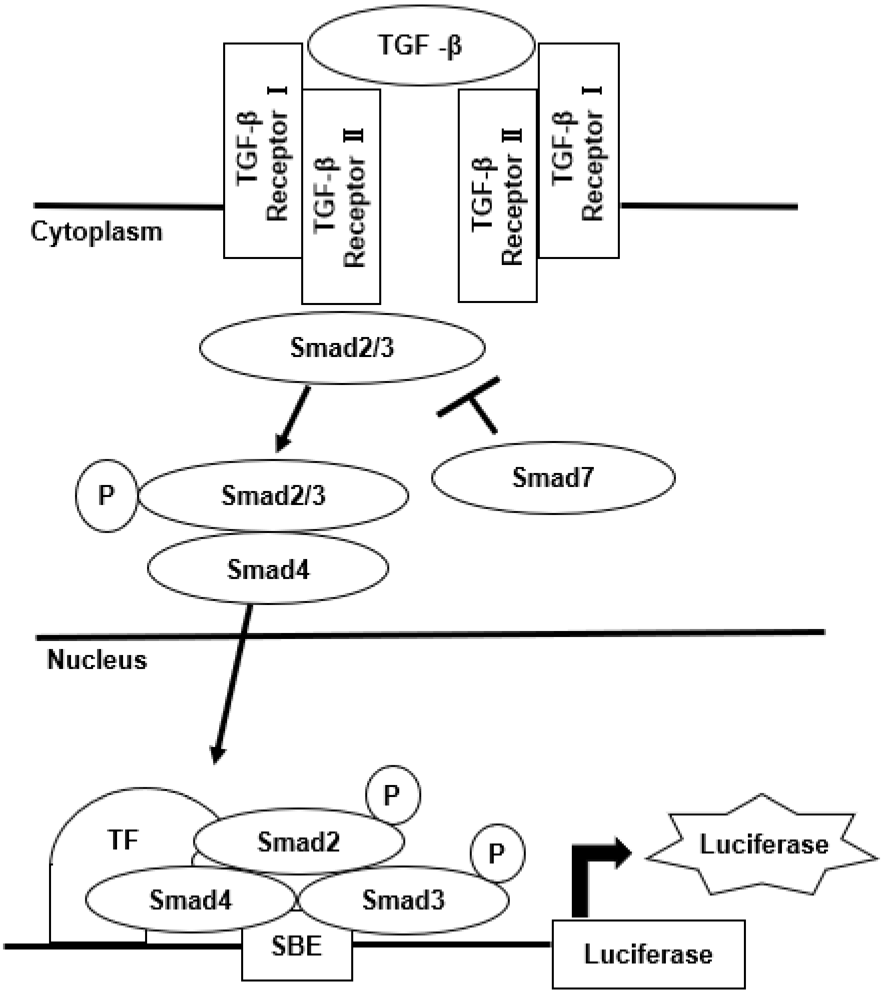

Supplement: Supplementary file 1 [file pharmaceuticals-16-00618-s001.zip › Figure S2.pdf]
