# Supplementary material for: Super Carbonate Apatite-miR-497a-5p Complex Is a Promising Therapeutic Option against Inflammatory Bowel Disease
Source: Pharmaceuticals (Basel). 2023 Apr 19;16(4):618. doi: 10.3390/ph16040618 (PMC10146939; doi:10.3390/ph16040618)

Supplementary Figure S3. Sequence of mmu-miR-497a-5p and has-miR-497-5p

TargetScan was used to identify a binding site at position of the Smad 7 mRNA 3' UTR that was complementary to the identical seed sequence of mmu-miR-497a-5p and hsa miR-497-5p. Note that hsa-miR-497-5p possesses two binding portions in the human Smad 7 mRNA 3' UTR.

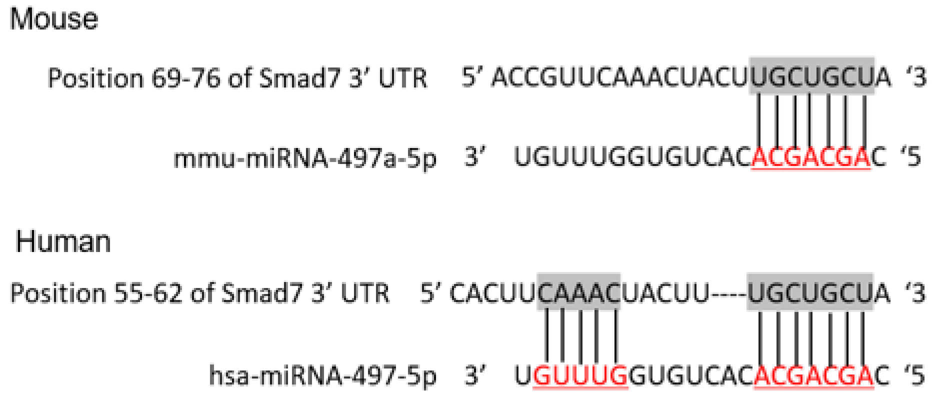

Supplement: Supplementary file 1 [file pharmaceuticals-16-00618-s001.zip › Figure S3.pdf]
