# Supplementary material for: Super Carbonate Apatite-miR-497a-5p Complex Is a Promising Therapeutic Option against Inflammatory Bowel Disease
Source: Pharmaceuticals (Basel). 2023 Apr 19;16(4):618. doi: 10.3390/ph16040618 (PMC10146939; doi:10.3390/ph16040618)

Supplementary Figure S4. HE staining of 1.5% DSS induced colitis mice

1.5% DSS was administered orally in drinking water to mice for 5 days. Mice were sacrificed on day 5. Strong inflammation was already observed in mucosa of rectum and distal colon. Scale bar: 100 mm

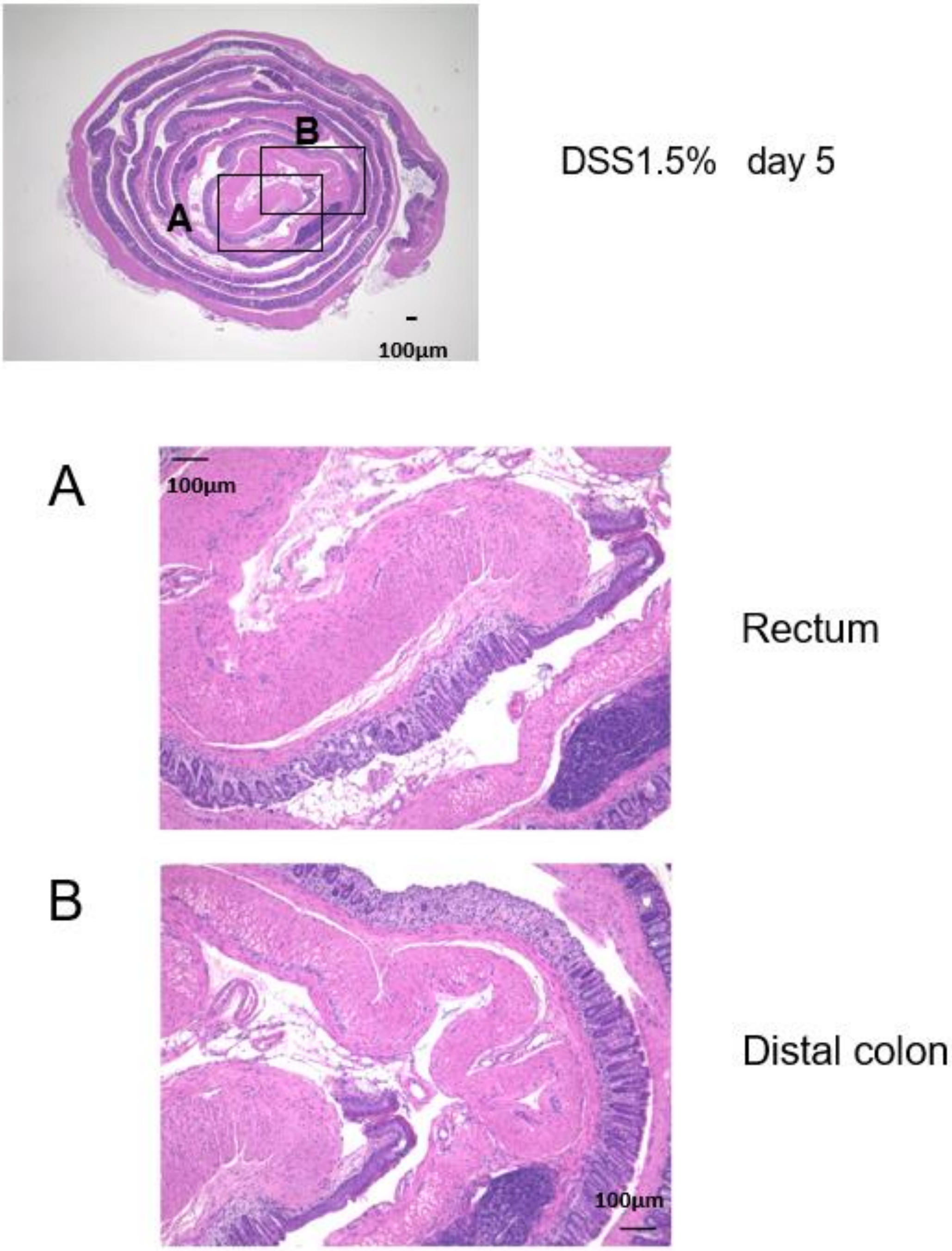

Supplement: Supplementary file 1 [file pharmaceuticals-16-00618-s001.zip › Figure S4.pdf]
