# Supplementary material for: Super Carbonate Apatite-miR-497a-5p Complex Is a Promising Therapeutic Option against Inflammatory Bowel Disease
Source: Pharmaceuticals (Basel). 2023 Apr 19;16(4):618. doi: 10.3390/ph16040618 (PMC10146939; doi:10.3390/ph16040618)
Supplement: Supplementary file 1 [file pharmaceuticals-16-00618-s001.zip › Supplementary Table S1.pdf]

Supplementary Table S1. Sequences of the miRNAs used in this study.

mmu miR-125a-5p- sense: 5’- UCCCUGAGACCCUUUAACCUGUGA-3’

mmu miR-125a-5p- antisense: 5’- UCACAGGUUAAAGGGUCUCAGGGA-3’

mmu miR-148a-3p- sense: 5’-UCAGUGCACUACAGAACUUUGU-3’

mmu miR-148a-3p- antisense: 5’-ACAAAAGUUCUGUAGUGCACUGA-3’

mmu miR-148b-3p- sense: 5’- UCAGUGCAUCACAGAACUUUGU-3’

mmu miR-148b-3p- antisense: 5’-ACAAAGUUCUGUGAUGCACUGA-3’

mmu miR-152-3p- sense: 5’- UCAGUGCAUGACAGAACUUGG-3’

mmu miR-152-3p- antisense: 5’-AGUCACGUACUGUCUUGAACC-3’

mmu miR-15a-5p- sense: 5’- CAGGCCAUACUGUGCUGCCUCA -3’

mmu miR-15a-5p- antisense: 5’-GUCCGGUAUGACACGACGGAGU -3’

mmu miR-16-5p- sense: 5’- UAGCAGCACGUAAAUAUUGGCG-3’

mmu miR-16-5p- antisense: 5’-AUCGUCGUGCAUUUAUAACCGC-3’

mmu miR-19a-3p- sense: 5’- UGUGCAAAUCUAUGCAAAACUGA-3’

mmu miR-19a-3p- antisense: 5’- UCAGUUUUGCAUAGAUUUGCACA-3’

mmu miR-19b-3p- sense: 5’-UGUGCAAAUCCAUGCAAAACUGA-3’

mmu miR-19b-3p- antisense: 5’-UCAGUUUUGCAUGGAUUUGCACA-3’

mmu miR-497a-5p - sense: 5'-CAGCAGCACACUGUGGUUUGUA-3'

mmu miR-497a-5p - antisense: 5'-UACAAACCACAGUGUGCUGCUG-3'

mmu miR-186-5p - sense: 5'-CAAAGAAUUCUCCUUUUGGGCU-3'

mmu miR-186-5p - antisense: 5'-AGCCCAAAAGGAGAAUUCUUUG-3'

mmu miR-195a-5p- sense: 5’- UAGCAGCACAGAAAUAUUGGC-3’

mmu miR-195a-5p- antisense: 5’-AUCGUCGUGUCUUUAUAACCG-3’

mmu miR-196a-5p- sense: 5’- UAGGUAGUUUCAUGUUGUUGGG-3’

mmu miR-196a-5p- antisense: 5’-AUCCAUCAAAGUACAACAACCC-3’

mmu miR-196b-5p- sense: 5’- UAGGUAGUUUCCUGUUGUUGGG-3’

mmu miR-196b-5p- antisense: 5’-AUCCAUCAAAGGACAACAACCC-3’

negative control-miRNA1-sense: 5'-UAA AUGUACCGCGCGUGGAGAGGAA-3'

negative control-miRNA1-antisense: 5'-UUCCUCUCCACGCGCAGUACAUUUA-3'

negative control-miRNA2-sense: undisclosed
